# Elasticity improves handgrip performance and user experience during visuomotor control

**DOI:** 10.1098/rsos.160961

**Published:** 2017-02-15

**Authors:** Michael Mace, Paul Rinne, Jean-Luc Liardon, Catherine Uhomoibhi, Paul Bentley, Etienne Burdet

**Affiliations:** 1 Department of Bioengineering, Imperial College of Science, Technology and Medicine, London, UK; 2 Division of Brain Sciences, Imperial College of Science, Technology and Medicine, London, UK; 3 Robotics Research Centre, School of Mechanical and Aerospace Engineering, Nanyang Technological University (NTU), Singapore

**Keywords:** handgrip interface, elastic, isometric, rehabilitation, grip force

## Abstract

Passive rehabilitation devices, providing motivation and feedback, potentially offer an automated and low-cost therapy method, and can be used as simple human–machine interfaces. Here, we ask whether there is any advantage for a hand-training device to be elastic, as opposed to rigid, in terms of performance and preference. To address this question, we have developed a highly sensitive and portable digital handgrip, promoting independent and repetitive rehabilitation of grasp function based around a novel elastic force and position sensing structure. A usability study was performed on 66 healthy subjects to assess the effect of elastic versus rigid handgrip control during various visuomotor tracking tasks. The results indicate that, for tasks relying either on feedforward or on feedback control, novice users perform significantly better with the elastic handgrip, compared with the rigid equivalent (11% relative improvement, 9–14% mean range; *p* < 0.01). Furthermore, there was a threefold increase in the number of subjects who preferred elastic compared with rigid handgrip interaction. Our results suggest that device compliance is an important design consideration for grip training devices.

## 1. Introduction

Interaction with the environment involves the exchange of forces while manipulation requires skillful force control and is a sensitive measure of motor condition [1,2]. For hand and finger training, this motivates *isometric training* based on force control without the need to support overt movements, for example using a force-sensing handle such as Tyromotion's Pablo device (www.tyromotion.com). Grip force control can also be used for human–machine interfaces and teleoperation applications, e.g. control of surgical robotics [3], and as a tool to study ergonomics and handgrip design [4]. Furthermore, grip strength is a pervasive clinical outcome supported by dynamometry-based isometric measurements (using the Jamar handgrip) [5,6]. Isometric training has been shown to enable the learning of force fields applied on virtual movements associated with the exerted isometric force and that this learning transferred to real (isotonic) movements [7,8]. However, such systems for isometric control or strength do not support the kinematic aspect of training which is an intrinsic part of manipulation and activities of daily living (ADLs) [9].

Grasping of objects involves grip aperture modulation and shaping of the hand, and often involves interaction with soft objects or manipulation [10]. This suggests that grip training should involve learning to shape one's hand across a range of joint angles similar to natural grasping tasks. Moreover, allowing the stretching of muscles can reduce collagen build-up in the joints and prevent further biomechanical issues such as contractures [11]. The MusicGlove system promotes finger individuation through finger tapping [12], while Neofect's Smartglove can measure overt movements of the digits using bend sensors [13], with both interfacing to virtual environments for training. A recent study in 12 chronic stroke patients with moderate hemiparesis comparing two weeks of movement-based training using the MusicGlove system to both isometric grip training and conventional therapy showed superior functional outcomes [12].

While skilful force control is critical to efficient manipulation, it may be helped by using additional joint position sensing. Indeed, proprioception can be divided into both static and dynamic components, and relies on various types of mechanoreceptors and skin afferents, including muscle spindles, Golgi tendon organs and skin stretch senses [14]. The different afferents respond in a variety of ways to different stimuli, for example muscle spindle receptors signal both the length and rate of change of muscles hence contributing to both the static and dynamic components [15]. The static component senses the stationary limb while the dynamic component involves the estimation of limb position and velocity during either volitionally generated active movements or passively induced motions. In fact, active movement itself as opposed to endpoint postures is thought to provide the greatest acuity for localization [16]. Therefore, elastic as opposed to isometric interaction will provide additional coordinated kinaesthetic information facilitating control and learning by playing a vital role during the planning and execution of voluntary movements [17,18]. A recent study comparing virtual learning based on isometric force information demonstrated the beneficial effect of additional elastic deformation on control and learning [19]. Damage to the neural circuits mediating proprioceptive function, e.g. due to an infarction in thalamic or parietal brain areas, can impair a patient's ability during goal-directed movement, prehension, accurate aiming, reaching and tracking movements [20,21]. This can occur in up to half of stroke patients and therefore technology that can stimulate proprioceptive feedback during active training are essential.

The vast majority of ADLs require a functioning hand. This explains why individuals with complete loss of movement capabilities select recovering arm and hand function as their number one priority for improving their quality of life [22]. Unfortunately, 77% of stroke survivors are affected by arm–hand weakness and poor control [23], while impaired hand function is also common in other neurological diseases such as cerebral palsy and multiple sclerosis. Hand function is also commonly impaired as a consequence of rheumatological and orthopaedic conditions such as symptomatic hand arthritis which is estimated to affect over 300 million worldwide [24]. The only intervention shown to improve arm function is repetitive, task-specific exercise, but this is limited by the cost and availability of physiotherapists [25,26]. To address this issue, we are developing affordable devices to promote independent training of hand function from the ward to the home. These simple devices provide accessible functional rehabilitation by working on improving hand function through the use of engaging virtual therapy games controlled via sensors. With such devices, it is possible to train hand functions through individuated finger movements or whole hand grip force control [27].

So how can one train using both force control and hand kinaesthesia with a passive device using no actuators? To manipulate objects such as a soft ball, one has to control the force which is coupled to motion through the object's elasticity. Similarly, we have created an elastic handle with a spring mechanism in series with a force transducer yielding force-sensing coupled with movement deformation. In a recent study, we showed that this sensitive mechanism enables even severely impaired patients to interact with a mobile tablet PC who would otherwise be unable to use such technology by conventional means, i.e. swiping, tapping and tilting [28].

This device has enabled us to study the effect of elasticity and resulting proprioceptive information on grip control. We have carried out a usability study with 66 healthy individuals, contrasting the elastic behaviour that this handgrip affords to isometric-equivalent interaction during visuomotor tracking tasks. We used two types of tasks, namely, one relying predominantly on feedforward information while the other relies on continuous sensory feedback. The digital handgrip and mobile-based virtual therapy platform used for this experiment are described in the next section, followed by the description of the visuomotor tasks and experimental protocols. The results presented in the following section reveal advantages of the elastic interaction over pure isometric information for grip control, alongside the influence of different factors on performance and preferences during the different interaction modalities.

## 2. Material and methods

### 2.1. Digital handgrip

**Figure 1.**
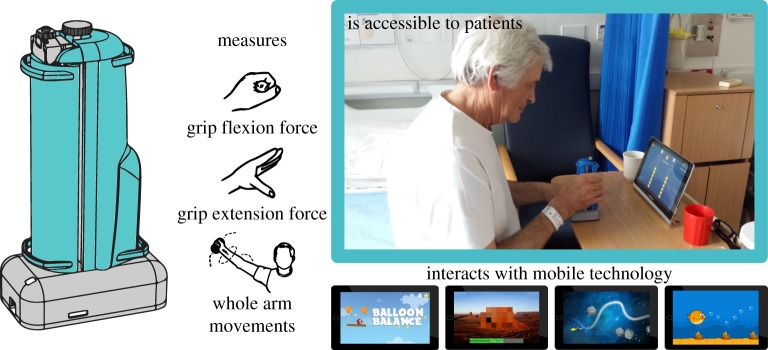
Overview of the interactive handgrip and mobile virtual training package including the motor behaviours the handgrip affords, alongside a photo of a patient using the digital handgrip and screenshots of some of the training games that have been developed.

We have developed an innovative digital handgrip that allows patient-led therapy and objective assessment of the upper limb. It comprises a force-sensing mechanism which enables the handgrip to deform elastically when squeezed [29]. Additional motion tracking sensors allow the simultaneous training and assessment of a variety of hand and upper-arm movements such as grasping and lifting an object. The handgrip interacts with specially designed app-based therapy games. These virtual therapy games are designed to be highly motivating, accessible to all ages and levels of cognitive function, and to automatically adapt to the ability of the individual. Figure 1 shows an overview of the handgrip concept including mobile-based virtual gaming therapy.

In this study, the internal structure of the handgrip consists of either the elastic force-sensing mechanism [30] or a completely stiff structure (isometric version) connected to a bidirectional ±5 kg 1 degree-of-freedom (d.f.) load cell (Phidgets 3133_0 micro load cell CZL635). Two external shells are mounted to one side of the load cell and the opposite side of the internal structure, respectively. These shells have been ergonomically designed to fit comfortably within the power grasp of a human hand with the rear shell located against the thenar eminence and the front shell in contact with the phalanges. Externally, there is no difference between the elastic and rigid handgrips. Grip extension can be facilitated with the use of straps (not shown).

The force-sensing mechanism uses a novel variable stiffness mechanical structure, coupling force and movement [30]. This mechanism is free from friction and backlash while supporting a range of bidirectional spring-like movements in grip flexion and extension. The device is highly sensitive and able to capture barely visible ‘flicker’ movements, which are often exhibited by individuals in the acute phase following physical impairment. It is worth noting that once completely compressed, the elastic handgrip will measure any additional grip force in an isometric manner up to the maximum force of the load cell (i.e. 50 N). The rigid version of the handgrip transmits the force exerted between the phalanges and thenar eminence entirely isometrically with no movement of the two shells occurring.

The handgrip sensitivity is only limited by the resolution of the 10-bit analogue-to-digital convertor (ADC) onboard the microcontroller unit (MCU), and noise introduced by the electronic circuitry. To further elevate the ADC resolution, an oversampling strategy has been implemented increasing the force resolution to 0.0288N/bit. The peak-to-peak noise of the load cell, pre-amplifier and MCU was measured as less than 50-bits post-oversampling which implies a sensitivity of less than 1.5 N. The sensitivity is further increased by digitally filtering the noise (pre-downsampling) using a fourth-order Butterworth filter with a cut-off frequency of 100 Hz. An inertial measurement unit (IMU, Bosch BNO055) is used to track the motion of the handgrip while a 10 mm coin vibration motor (Precision Microdrives 310-103), which is located under the rear shell, enables vibro-tactile stimulation during handgrip interaction (although both these functions are not used in this study). The electronics (MCU, pre-amplifier, IMU, vibrator drive circuits, bluetooth transmitter and battery) are housed in the base of the handgrip, which enables capture and wireless transmission of the force and motion data to a tablet PC at a sampling rate of 50 Hz. In this study, only the force data are analysed. Table 1 summarizes the system characteristics associated with the handgrip devices and the values chosen for this study. For the remainder of this paper, we refer to *soft* interaction as that provided by the novel elastic force-sensing mechanism which flexes when squeezed (i.e. measures force alongside movement) and *rigid* interaction as that provided by the stiff structure which does not flex when squeezed (i.e. only measuring the isometric force).

**Table 1.** System characteristics and salient values associated with both elastic and rigid handgrip devices.

system characteristic	possible values	values used
range	±50 N	0–20 N
resolution	0.0288 N bit^−1^	0.0288 N bit^−1^
sensitivity	<1.5 N	<1.5 N
ROM^a^	±10 mm	0–10 mm (flexion)
grip aperture (unloaded)	55–75 mm	65 mm
compliance^a^	0.06–0.5 mm N^−1^	0.2 mm N^−1^

^a^Applicable to the elastic handgrip only.

### 2.2. Visuomotor control tasks

**Figure 2.**
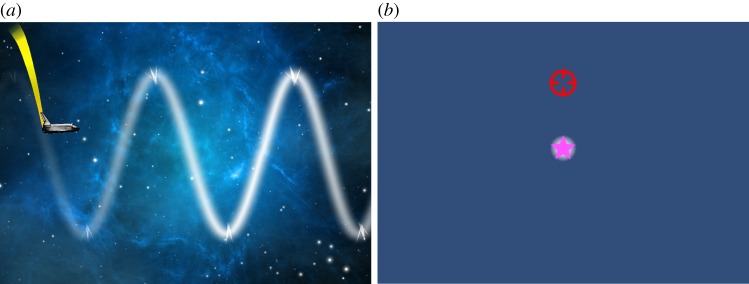
Screenshots showing the feedforward task (‘SpaceWay’ game; (*a*)) and feedback task (‘StarShooter’ game; (*b*)).

Two different tasks, corresponding to two tracking conditions, are used to test feedforward and feedback control, respectively. In both tasks, the subject controls the vertical position of an on-screen cursor proportionally to their grip force to track a continuous reference trajectory. In the *feedforward condition*, motion planning is promoted by scrolling the background horizontally at a constant speed relative to the cursor. At any point in time, this enables the subject to see the reference trajectory up to 5 s before they have to react to it and thus they have time to plan the necessary grip force action (i.e. squeeze or relax) in advance of the action being required. In the *feedback condition*, the subject has to follow the cursor but has no visible reference trajectory. This is achieved by moving a reference target centrally in the vertical plane based on an unknown but continuous reference path. The subject is tracking this path by trying to align the cursor with the target at all times. As the subject is unable to plan the required action, they will rely on the instantaneous visual information alongside knowledge of their current grip force state, perhaps relying more on fast kinaesthetic sensing rather than on slower visual feedback [31,32]. Figure 2 shows the visual information presented during each of the tasks. Both tasks have been gamified, with the feedforward task defined through a ‘SpaceWay’ game whereby the cursor is a spaceship and the reference trajectory is shown as a path of space dust to follow. The feedback task is defined through a ‘StarShooter’ game whereby the cursor is a crosshairs and the target is a moving purple star.

**Figure 3.**
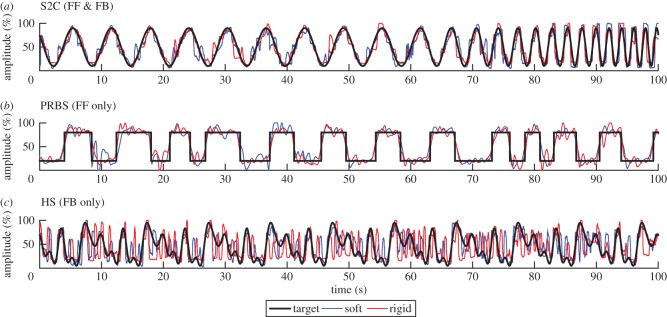
Sample trajectories from one representative subject tested during the visuomotor control tasks alongside example data from both soft (blue) and rigid (red) interactions. (*a*) Sin-to-chirp (S2C) waveform tested under both feedforward and feedback conditions, (*b*) PRBS waveform tested under the feedforward condition only and (*c*) HS waveform tested under the feedback condition only.

Three reference trajectories have been used to test the tracking abilities across subjects and devices (elastic or rigid version). The trajectories tested are a sin-to-chirp (S2C) function, a pseudo random binary sequence (PRBS) and a harmonic series (HS). Figure 3 shows the target trajectories alongside example data from a subject using either the elastic or rigid handgrip. The S2C trajectory was tested on both the feedforward (FF) and feedback (FB) tasks, while the PRBS trajectory was tested on the FF task only and the HS trajectory was tested on the FB task only. Table 2 summarizes which subject groups (T1 or T2) were tested with which task condition (feedforward or feedback).

**Table 2.** Overview of the trajectories performed by the two different subject groups (T1 = 34 subjects and T2 = 32 subjects).

order tested	group T1 (feedforward)	group T2 (feedback)
first	S2C	S2C
second	PRBS	HS

For each subject group and device tested, the reference signals were kept fixed and lasted for approximately 2 min. To follow the reference trajectory, the subject was required to increase their grip flexion force which proportionally increases the vertical cursor height on the screen. Releasing their grip force (grasp relaxation) will allow the cursor to return back towards the bottom of the screen.

### 2.3. Participants

Thirty-four healthy adults ranging from 20 to 77 years in age (mean ± 1 s.d.: 43.0 ± 17.7 years, gender: 15F/19M) were recruited for the feedforward condition, 32 healthy adults ranging from 17–67 years in age (37.8 ± 15.0 years, gender: 18F/14M) for the feedback condition. Participants had no known impairment, and were all right-handed with an average handedness score of 79.2 ± 38.2 based on the Edinburgh Handedness Inventory. There were no significant differences in the ages, gender or handedness of the two healthy subject groups (age: *p* = 0.21, gender: *p* = 0.33, handedness: *p* = 0.51). The two groups represent a diverse cross-section of society with a uniform gender distribution and ages spanning six decades. Half the subjects had an age of 40 years or older thus age-matching to many neuromotor impairments such as stroke. Table 3 summarizes this information in more detail.

**Table 3.** Average characteristics describing the two different subject groups (used during the feedforward and feedback tasks) and combined together.

	group T1 (FF)	group T2 (FB)	combined (FF and FB)
No. subjects	34	32	66
age (years)	43.0 ± 17.7	37.8 ± 15.0	40.4 ± 16.6
gender	15F/19M	18F/14M	33F/33M
handedness	85.5 ± 20.9	67.9 ± 56.6	79.2 ± 38.2

### 2.4. Experimental protocol

None of the subjects had previous experience of the device or tracking task. To remove any learning bias, an SR/RS block protocol was employed whereby half of the subjects started with the soft (S) or rigid (R) device. The subjects were seated comfortably at a table with a 10-inch tablet PC situated approximately 30 cm in front of them. The device was connected wirelessly to the tablet PC. The subjects were initially instructed to hold their first assigned device in their right hand in a comfortable and consistent position, and to use this same position across all trials. They then performed 2 min of the S2C tracking task, followed by a 1 min rest and then 2 min of the PRBS (FF group) or HS (FB group) tracking task. They were asked to answer six questions on a questionnaire pertaining specifically to that device after completing both trajectories. The same steps were then repeated for the alternative handgrip device. Finally, two additional questions were answered on the questionnaire.

### 2.5. Questionnaire

The following six questions were answered by each participant, once for each device, directly following a device trial block (S or R). Each question was rated on a discrete five-point Likert scale.

— Q1: How well do you think you *performed*?
— Q2: Did you *enjoy* using the device?
— Q3: Did you experience any *discomfort* while using the device?
— Q4: Do you think you *improved* whilst using the device?
— Q5: Did your hand feel *fatigued* after using the device?
— Q6: Did you feel in *control* when using the device?

Two additional questions were asked at the end of the session which were also rated on five-point Likert scales.

— QG: How often do you play computer, tablet or smartphone *games*?
— QP: Which device did you *prefer* to use?

### 2.6. Data analysis

The ability for an individual to track a target trajectory (*y*) was measured using the root mean squared (RMS) error. Initially, the subject's response ($\hat{y}$) during a trial is aligned to *y* using the position of maximum cross-correlation. This removes any systematic error due to the cursor not being displayed as a single-point onscreen. For each trial, the error is calculated in a moving window, enabling the minimum error across windows to be extracted. Consequently, this metric describes the best error achieved for a given period of tracking and mitigates any short-term or sporadic artefacts e.g. due to accidental movements, lapses in subject concentration, or fatigue. The RMS error (*E*) is computed at each time step using

2.1 $$E_{n}=\sqrt{\frac{1}{W}\overset{n+W/2}{\underset{k=n-W/2+1}{\sum }}{\left({\hat{y}}_{k}-y_{k}\right)}^{2}},$$

where *N* is the total number of samples in the trial, *W* is the length of the window and *E_n_* is calculated in the range [W/2+1,W/2+ 2,…, N−W/2] to prevent boundary effects. The final performance metric (for a given trial and window length), defined as the minimum moving error (MME), is given by

2.2 $$\text{MME}=\min_{n}(E_{n}).$$

### 2.7. Statistical tests

To test significant differences, non-parametric Mann–Whitney *U* (MWU) tests were chosen due to the relatively small sample sizes (*N* = 32/34) and underlying distributions associated with the variables of interest. In the case of the performance data (i.e. MME), the distribution is skewed and has a fixed [0,100%] range. The questionnaire data are discrete, but ordinal, and therefore can be analysed using the same assumptions. For these tests, a *χ*^2^-test was not appropriate due to the expected small frequencies of some of the entries. Paired tests were used to compare soft and rigid interactions, while an equivalent unpaired MWU test was employed while testing across different populations (i.e. feedforward versus feedback task conditions). Only individual pairwise comparisons (i.e. no multiple comparisons) were required.

Interactions between performance differences, preference and device testing order were analysed using Fisher exact (FE) testing under the null hypothesis of pairwise independence. Differences in performance between the two handgrip types was computed as a binary variable indicating whether the MME was better for soft or rigid device interaction. Preference was extracted from the questionnaire data as a binary variable indicating whether a particular subject preferred the rigid or soft device (with neutral data points ignored). The device testing order is already a binary variable indicating which device was tested first. Thus, all three variables can be treated as binary and therefore categorical data.

Linear regression was used to analyse relationships between age and performance for each device, task and trajectory. Both ordinary least squares (OLS) and robust least squares (RLS) were used. RLS uses an iterative reweighted least-squares method based on a bisquare function allowing outliers to be either ignored or proportionally weighted when computing best-fit lines.

To test the effects of gaming experience on performance, a non-parametric Kruskal–Wallis (KW) test was performed. It is worth noting that this test makes the assumption that the gaming experience data are categorical rather than ordinal. The effect of age and gaming experience on preference was analysed using FE tests. A binary age variable indicating whether a subject was young (less than or equal to 40 years) or old (greater than 40 years), a binary gaming experience indicating whether a subject was experienced (plays games weekly or more) or inexperienced (plays games monthly or less), and a binary preference variable (calculated as before) were used in this analysis. As these tests proved insignificance (see Results section), no further (post-hoc) significance testing was required.

## 3. Results

### 3.1. Performance

**Figure 4.**
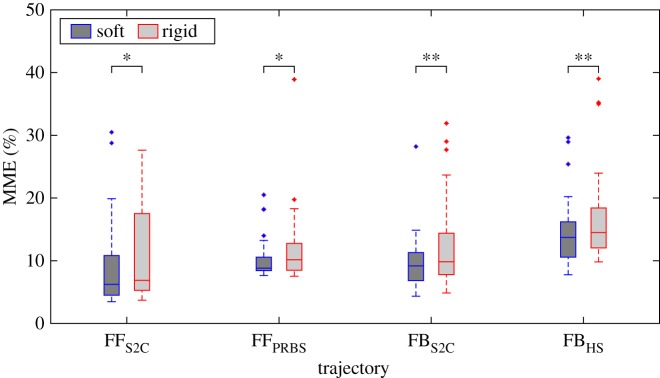
Comparison of minimum moving error between soft and rigid interaction for the four tasks. FF indicates feedforward and FB indicates feedback conditions. S2C is the sin-to-chirp, PRBS is the pseudo random binary sequence and HS is the harmonic series trajectories. Asterisk indicates a significant difference of *p < 0.05, **p < 0.01.

Figure 4 shows comparative box plots of the MME for the two devices tested (soft or rigid) across the four task types (FF_S2C_, FF_PRBS_, FB_S2C_ and FB_HS_). For each task, a paired MWU test was used to infer whether the median difference in performance was significant and is shown for each of the four tasks. The best error was calculated in a 30 s analysis window (i.e. *W* = 30), which was felt to be an adequate compromise between length and rejection of spurious events. Appendix A describes in more detail reasons behind this choice of W alongside sensitivity analysis highlighting that the performance differences between the devices is not strongly affected by the choice of *W*.

The relative pairwise difference between the MME across the two devices are FF_S2C_ = 10.3 ± 27.1%, FF_PRBS_ = 8.8 ± 17.9%, FB_S2C_ = 14.4 ± 26.4% and FB_HS_ = 11.4 ± 17.7% (mean ± s.d.), indicating that the soft handgrip enabled better performance than the rigid equivalent. This relative MME difference is normalized by the MME of the rigid device to highlight the relative improvement for the soft interaction compared to the more conventional rigid handgrip interaction. This difference is consistent across all conditions (*p* < 0.05) with the feedback condition showing high significance (*p* < 0.01) when tested using a paired MWU test. Across all tasks, the relative MME difference is 11.2 ± 22.6% in favour of soft interaction.

### 3.2. Questionnaire

**Figure 5.**
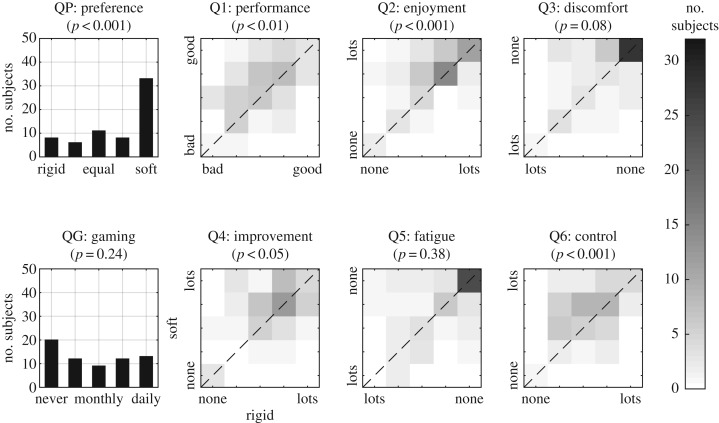
Results from the questionnaire shown as univariate five-point histograms (QP, QG; left column) or five-point joint frequency distributions (Q1 … Q6).

Figure 5 shows the collated results for the eight questions across both tasks, corresponding to 66 subjects. Results of the device preference (QP panel) and gaming experience (QG panel) questions are displayed as individual five-point histograms and highlight the distribution across the answers given. The six questions (panels Q1 … Q6) show joint frequency distributions between the paired answers associated with both the soft and rigid handgrips. The axes of Q3 and Q5 have been inverted so that for all six questions, negatively perceived answers are presented towards the bottom-left while positive answers are towards the top-right. For each question, a paired MWU test was performed to highlight if the median of the difference between soft and rigid answers (Q1 … Q6) or the distribution itself (QG, QP) is significantly different from zero, with the results shown above each plot.

Subjects found the soft force measuring handgrip advantageous to the rigid one due to several factors. Over 62.1% of subjects preferred the soft handgrip, 21.2% preferred the rigid device and approximately 16.7% showed no preference (QP, *p* < 0.001). Subjects thought they performed better (Q1, *p* < 0.01), enjoyed interacting more (Q2, *p* < 0.01), improved more (Q4, *p* < 0.05) and had better control (Q6, *p* < 0.01), when using the elastic handgrip. Subjects felt they had the same level of discomfort and fatigue for both device modalities, with over 28/66 subjects experiencing no fatigue or discomfort at all.

### 3.3. Interactions

An analysis was performed to investigate the interplay between performance, preference and other experimental variables such as device testing order, age and questionnaire responses, yielding the following results.

#### 3.3.1. Gaming experience has no effect on performance or device preference

KW tests revealed that, for both rigid and soft handgrip interaction, there were no significant differences in performance corresponding to the level of gaming experience (*p *> 0.05 across all tasks and interaction modalities). FE tests also showed that, for both feedforward and feedback conditions, gaming experience did not have an effect on device preference (FF: *p* = 0.67 and FB: *p* = 1.0).

#### 3.3.2. Age affects performance but not differences in interaction modalities or device preference

FE tests revealed that, for both feedforward and feedback conditions, age does not have an effect on device preference (FF: *p* = 1.0 and FB: *p* = 0.61). Unpaired MWU revealed that performance differences between soft and rigid interaction for younger (less than 40 years) and older (greater than or equal to 40 years) subjects were insignificant across all tasks (FF_S2C_: *p* = 0.76, FF_PRBS_: *p* = 0.19, FB_S2C_: *p* = 0.31 and FB_HS_: *p* = 0.83).

**Figure 6.**
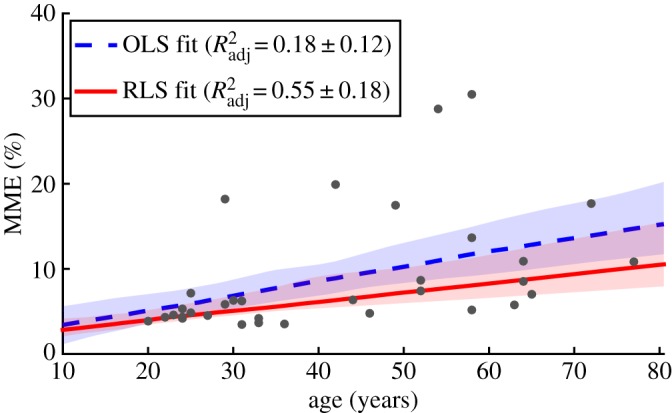
Scatterplot of performance (MME) versus age during the FF_S2C_ task using soft interaction. Also shown are both OLS and RLS fits and associated *R*^2^_adj_ values.

To understand the effect that age has on performance, linear models were computed between the age of the subjects (continuous-independent variable) and the MME during each task (continuous-dependent variable). To mitigate the effects of outliers, RLS alongside OLS was used. Figure 6 shows example (OLS and RLS) linear fits alongside the *R*^2^_adj_ goodness-of-fit values during the FF_PRBS_ task; 95% confidence intervals are shown computed through bootstrapping (using 1000 resamples). Across all eight conditions (four tasks and both interaction modalities), the average *R*^2^_adj_ value was higher for the RLS method (OLS: *R*^2^_adj_ = 0.22 ± 0.17 and RLS: *R*^2^_adj_ = 0.31 ± 0.11) highlighting that the presence of outliers was likely in the datasets. Therefore, further analysis was only performed using the RLS method.

**Figure 7.**
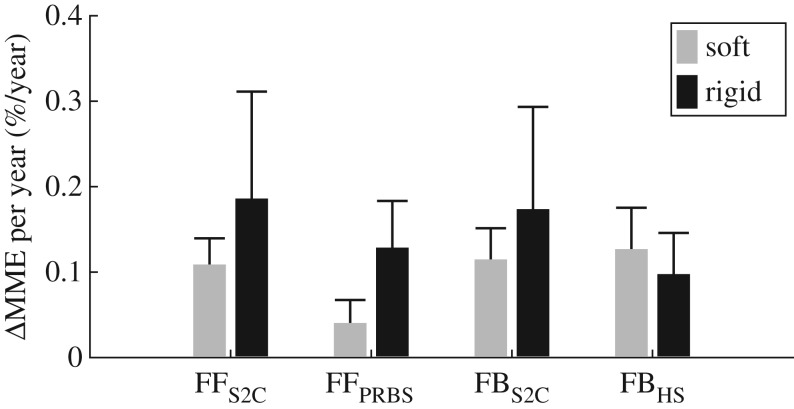
Comparative bar graphs highlighting the distribution of the slopes for age versus performance (median ± IQR) comparing soft and rigid interaction. Slopes were computed using RLS linear fits.

The average slope of the RLS linear model was positive (RLS: *slope* *=* 0.12 ± 0.05%/year), highlighting that regardless of the interaction mode or task, MME does increase with age. Figure 7 shows the median and interquartile range (IQR) of each slope, computed using the bootstrapped data, contrasting rigid and soft device interaction across all tasks. These results suggest that soft interaction may give less age-related performance deficits for certain types of task (i.e. FF_S2C_, FF_PRBS_ and FB_S2C_). However, these differences were found to be insignificant (FF_S2C_: *p* = 0.50, FF_PRBS_: p = 0.10, FB_S2C_: *p* = 0.36 and FB_HS_: *p* = 0.98).

#### 3.3.3. Do performance differences between the rigid and soft handgrips have an effect on device preference?

FE tests revealed that for three of the four trajectories, device preference was not associated with differences in performance (FF_S2C_: *p* = 0.20, FF_PRBS_: *p* = 0.39, FB_S2C_: *p* = 0.07 and FB_HS_: *p *< 0.05). Specifics of this association in the FB_HS_ task highlighted that the majority of subjects who performed better with a particular device preferred that device (soft: 16/20, rigid: 4/5).

#### 3.3.4. Subjective sense of performance, enjoyment and control influences the preference for soft handgrip interaction

FE tests revealed that, across both task types (FF and FB), device preference was dependent upon differences in responses for three of the six questions. Specifically, preference was influenced by a different perception of performance (question Q1: *p* < 0.001), enjoyment (Q2: *p* < 0.05) and control (Q6: *p* < 0.01). It was not influenced by the other questions regarding discomfort (Q3: *p* = 1.0), improvement (Q4: *p* = 0.12) and fatigue (Q5: *p* = 0.63) for which both soft and rigid handgrip devices were found to provide similar advantages (figure 5). Further analysis of the specifics of this interaction highlighted that these associations were because subjects who felt they performed better with a device (soft: 28/28, rigid: 6/8), enjoyed using that device more (soft: 18/21, rigid: 3/4) and felt they controlled the device better (soft: 27/30, rigid: 5/7) usually preferred that device. Similar analysis of interactions between actual performance differences and questionnaire responses found no significant associations.

#### 3.3.5. The order in which the devices are tested affects the preference of and performance with the soft device

FE tests revealed that for two of the tasks, the device testing order (i.e. soft followed by rigid or vice versa) did have an effect on performance (FF_S2C_: *p* = 0.16, FF_PRBS_: *p* < 0.05, FB_S2C_: *p* < 0.01 and FB_HS_: *p* = 0.44). Further analysis of the specifics of these associations highlighted that they were due to subjects who started with the soft device performing similarly (FF_PRBS_: 7/17, FB_S2C_: 8/17) while for subjects who started with the rigid device, the majority of subjects performed better with the soft device (FF_PRBS_: 15/17, FB_S2C_: 15/15) during these two tasks. This indicates that practising with the soft device helped using the rigid one, but not the converse.

FE tests revealed that device preference was also dependent on the order that the devices were tested, showing significance for both tasks (FF and FB: *p* < 0.05). Further analysis of the specifics of this association highlighted that it was due to subjects who used the soft device first being (13/25) undecided in their preference, while subjects who used the rigid device first were (28/30) in favour of the flexible device.

## 4. Discussion

During all the visuomotor tracking tasks, subjects showed an 11% relative improvement in performance when using the soft compared with the rigid handgrip. Moreover, there was a threefold greater preference for the soft interaction with subjects feeling they could perform better, enjoyed it more and had increased control with it. Our results support neurophysiological and behavioural studies suggesting that kinaesthesia enhances motor control [17,18,33–35], and shows for the first time that functional grip trainers can facilitate performance and subjective experience by being elastic rather than rigid.

In our experiments, overall performance in the feedback task was reduced relative to the feedforward task (figure 4), indicating that generally subjects found this type of task more challenging. Larger errors for the FB_HS_ task can potentially be attributed to the lack of movement planning afforded by the feedback condition which makes (pseudo) random trajectories especially challenging [36]. Differences between the interaction modes in terms of relative MME error were also more pronounced for the feedback condition, while only the FB_HS_ task exhibited associations between performance and preference.

Differences in the availability of somatosensory feedback is the major difference between the soft and rigid controllers. Specifically, isometric control lacks significant contributions from proprioception due to the lack of movement. Zhai *et al.* [33,34] hypothesized that it was this additional sensory information that enabled novice users to have superior performance when comparing 6 d.f. control using an elastic or rigid upper limb interface. In comparison to our study, this work involved only proximal arm control (i.e. not functional grip force control), used fewer and only young subjects and incorporated only a single feedback-type condition. Their subjective evaluations also highlighted that isometric control was both more difficult and fatiguing during continuous tracking tasks. With more training (greater than 20 min), the performance of the two input devices converged to a similar level with the authors suggesting that the manipulation shifted from closed-loop to a more open-loop behaviour. In the feedback condition, we hypothesize that the additional kinaesthetic feedback provided by the soft handgrip would be especially useful, which may explain the difficulty in dealing with the feedback task observed in the isometric condition. A similar positive effect of the addition of elasticity was observed in a recent study in which virtual learning based on isometric force information and an inverse dynamic model of the arm during constrained movements was improved by physical compliance and led to better learning [19].

The handgrip testing order was found to influence the performance and preference between the two devices. The number of (soft followed by rigid handgrip) and (rigid followed by soft handgrip) test blocks were randomly assigned and of equal number, ensuring that overall effects were still valid. Interestingly, subjects who started with the rigid handgrip were significantly superior with and unanimously preferred the soft device. *A priori*, this effect might be interpreted as an effect of more training before using the second, soft device, and also by the fact that the subjects had just used this device allowing them to clearly focus on its qualities. However, this hypothesis is contradicted by the distinctly different results obtained for the subjects who ended with the rigid device but did not necessarily prefer it. This dissymmetry of appreciation of the two devices depending on the order they had been practised with suggests a clear preference and performance improvement with the soft handgrip.

The influence of age on performance highlighted an age-related reduction in grip control capabilities for both feedforward and feedback-type visuomotor tasks. Similar correlations between age and decline in (isometric) grip force control have been found in previous studies (e.g. [37–39]). Older subjects generally had a smaller error when using the soft device, suggestive that the additional kinaesthetic information it provides may be useful for older subjects during grip control of visual tracking tasks. Despite this, experience was found to have no relation to performance indicating that the handgrip and tasks defined in this study can be used by anyone regardless of previous exposure to games and associated skill levels involved. Future studies will investigate a similar question regarding elastic versus rigid handgrip interaction in patients affected by arm–hand weakness and poor control, e.g. due to stroke.

In conclusion, our results demonstrate that, regardless of age and experience, coupling force and position through an elastic structure has positive effects in terms of performance and subjective experience during grip force control. We hypothesize that this advantage is due to the coupled movement, providing additional sensory information including (dynamic) proprioceptive and cutaneous feedback. Therefore, device elasticity is an important consideration when designing new grip measurement devices and further enables the training of hand dexterity and strength alongside functional movements. This should be considered when designing a handgrip for training and rehabilitation, or more generally as a human–machine interface.

## Figures and Tables

**Figure 8.**
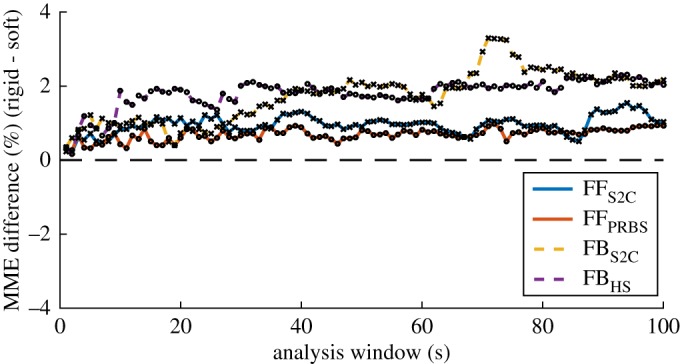
Relative MME difference between elastic and rigid handgrip interactions across different window sizes.

## Data accessibility

Our data are deposited at the Dryad Digital Repository: http://dx.doi.org/10.5061/dryad.m68q0 [40].

## Appendix A. Sensitivity analysis

Sensitivity to the analysis window (*W* in equation (2.1)) was examined to check that its choice would not affect interpretation of the results. As the main outcome is the (paired) MME differences between soft and rigid handgrip control, this was tested against increasing *W*. Figure 8 shows the median error difference for the four trajectories (FF_S2C_, FF_PRBS_, FB_S2C_ and FB_HS_) across all subjects as a function of *W* (computed at 1 s intervals). It can be seen that for any choice of *W* the error difference remains in favour of the soft handgrip and that after 10 s these differences are relatively stable. Therefore, *W* is chosen as 30 s providing a good compromise between length and rejection of spurious events. An additional benefit of performing this type of analysis is that the effects that duration (and time) have on the error differences can be seen. For example, between 70 and 80 s, there seems to be a relatively big jump in the FB_S2C_ error difference which could be caused by incorporating the transition of the reference path from a regular sinusoid to a time-dependent chirp signal. The instantaneous error estimation at this transition is more likely to be higher due to a change in the required control and lack of motion planning capabilities for the FB tasks, and (on average) seems to be accommodated better using soft handgrip control.
